# A Grey Wolf Optimization-Based Track-Before-Detect Method for Maneuvering Extended Target Detection and Tracking

**DOI:** 10.3390/s19071577

**Published:** 2019-04-01

**Authors:** Bo Yan, Xu Yang Zhao, Na Xu, Yu Chen, Wen Bo Zhao

**Affiliations:** 1 School of Aerospace Science and Technology, Xidian University, 266 Xinglong Section of Xifeng Road, Xi’an 710126, China; boyan@xidian.edu.cn (B.Y.); YuCheng@stu.xidian.edu.com (Y.C.); 2 School of Electronic Engineering, Xidian University, 266 Xinglong Section of Xifeng Road, Xi’an 710126, China; xyzhao07@stu.xidian.edu.cn; 3 School of Life Sciences and Technology, Xidian University, 266 Xinglong Section of Xifeng Road, Xi’an 710126, China; nxu_1994@stu.xidian.edu.cn

**Keywords:** track-before-detect, extended target tracking, grey wolf optimization

## Abstract

A grey wolf optimization-based track-before-detect (GWO-TBD) method is developed for extended target detection and tracking. The aim of the GWO-TBD is tracking weak and maneuvering extended targets in a cluttered environment using the measurement points of an air surveillance radar. The optimal solution is the trajectory constituted by the points of an extended target. At the beginning of the GWO-TBD, the measurements of each scan are clustered into alternative sets. Secondly, closely sets are associated for tracklets. Each tracklet equals a candidate solution. Thirdly, the tracklets are further associated iteratively to find a better solution. An improved GWO algorithm is developed in the iteration for removal of unappreciated solution and acceleration of convergence. After the iteration of several generations, the optimal solution can be achieved, i.e. trajectory of an extended target. Both the real data and synthetic data are performed with the GWO-TBD and several existing algorithms in this work. Result infers that the GWO-TBD is superior to the others in detecting and tracking maneuvering targets. Meanwhile, much less prior information is necessary in the GWO-TBD. It makes the approach is engineering friendly.

## 1. Introduction

Maneuvering weak target detection and tracking is always a challenging problem in modern radar systems. Its purpose is to detect, track and identify targets from sequences of measurements and clutter. For the increased resolution of modern radar, radars are able to receive more than one measurement per time step from different corner reflectors of a single target. Various algorithms have been developed for extended target detection and tracking. The algorithms are mainly fall into two categories: extended target probability hypothesis density (ET-PHD) filters [1,2,3,4,5] and track-before-detect algorithms [6,7,8,9,10]. 

ET-PHD-based algorithms [1,2,3,4,5] are capable of estimating the target extent and measurement rates as well as the kinematic state of the target. Correct partitions are significant to achieve good tracking performance. Therefore, various partitioning methods have been also developed. Reference [1] shows the application of distance partitioning algorithms for the partitions of the measurement set in PHD filters. Distance thresholds are insufficient to generate enough partitions for the correct partition. Increases of unappreciated partitions make the extended target tracking process computationally intractable. Therefore, a novel fast partitioning algorithm with fuzzy adaptive resonance theory (ART) model for the ET-PHD filter is proposed in [11]. Then, affinity propagation clustering is introduced into the measurement partitioning for extended target tracking in [12]. For the presence of clutter measurements, extended target which only generates a few measurements is hard to be detected. Track-before-detect algorithms [6,7,8,9,10] are superior in detecting and tracking weak targets for taking full merits of multiscan. Track-before-detect algorithms mainly have three implementations: particle filter based track-before-detect (PF-TBD) [6], dynamic programming based track-before-detect (DP-TBD) algorithms [7,8] and Hough transformation based track-before-detect (HT-TBD) [9,10]. However, drawbacks still exist. In PF-TBD, the probability distribution of particles is changing when the target is maneuvering. A straight-line constant-velocity mobility model is assumed for the target because the HT is designed to extract straight-line target trajectories in the Cartesian data. Therefore, the detection rate and tracking precision can be greatly deteriorated when the target is maneuvering. DP-TBD maintains track trees for incompatible tracks and discards the unreliable tree branches (track hypotheses) formed on last scans. Enumeration of hypotheses is impractical for the real-time application as the number of hypotheses exponentially increases with a linear increases in the depth of the hypotheses. Above discussion implies that existing methods [1,2,3,4,5,6,7,8,9,10,11,12] are insufficient to detect and track the maneuvering weak extended targets. 

In recent decades, there is a significant growing attention for nature-inspired computation, in which the two most popular algorithms are Swarm Intelligence (SI) and Evolutionary Algorithms (EAs). SI, like the Ant Colony algorithm (ACO) [13], Artificial Fish Swarm (AFS) [14] algorithm, Artificial Bee Colony algorithm (ABC) [15] and Particle Swarm Optimization (PSO) [16] algorithm, is inspired by animals’ foraging behavior. EAs, such as the Genetic Algorithm (GA) [17], and Evolutionary Programming (EP) [18] are inspired by natural selection and the survival of the fittest in the natural world. Similar to the existing nature-inspired algorithms, a new mimic algorithms on the basis of the behavior of grey wolves was proposed in the last few years. The grey wolf optimization (GWO) algorithm [19] has been clearly proved to be better than the optimization in [13,14,15,16,17,18]. It is worth noting that GWO algorithm has been used to solve the model predictive control formulation in planning the optimal trajectories of multi-UAVs [20]. The result [20] also showcases that the GWO is superior to the several other optimization methods for its strong search ability. However, the GWO algorithm, or other SI and EA-based methods, to the best of our knowledge, has previously not been used in a framework for tracking maneuvering extended targets, in the presence of missed detection and clutter. Although the GWO algorithm has been widely used in various engineering problems, no such a GWO-TBD for extended target tracking problems has ever been developed. Therefore, GWO is integrated into the track-before-detect algorithm to achieve better trajectory, providing what we call “GWO-TBD”. 

In the GWO-TBD, a track-before-detect framework is utilized for making full use of multi-scan merits. This is beneficial to weak target detection. Meanwhile, the track-before-detect framework is able to perform target detection, data association, track initiation, and track maintenance at the same time. Its main limitations are related to the computational complexity and memory requirements for all possible association. Two strategies are developed to mitigate the problem. Firstly, tracklets are built. Each tracklet is potentially originated from the real trajectory. Secondly, GWO [19] is applied here to explore the most appropriate association of points and tracklets. Enormous calculation and memory requirements can be saved in finding the optimal trajectory for the four merits of GWO: simplicity, flexibility, derivation-free mechanism, and local optima avoidance. 

The wolf (candidate solution) in the GWO-TBD is a potential trajectory consisting of measurements (points). The value of the fitness function is the probability that the candidate solution is the trajectory of an extended target. The α wolf (optimal solution) can be achieved iteratively by the GWO algorithm. Meanwhile, different from other previous GWO methods, some necessary modifications are essential to match the target tracking problem. Firstly, a tracklet fusion stage is added in the iteration, like the crossover in GA, for generating better candidate solutions. Secondly, the target detection is more likely a point selection issue where the search space is modelled as an n-cube. It is important to assign for every wolf a set of coordinates that indicate if the selected points will belong to the final optimal trajectory. The GWO-TBD algorithm has four merits.

Firstly, unlike current ET-PHD-based filters that use only the data present in the current scan, our GWO-TBD uses multiple scans (including the current scan and some past scans). A higher detection rate can thus be achieved. Secondly, the GWO-TBD has fewer parameters to adjust. It makes the approach more flexibility and engineering-friendly. Thirdly, population-based metaheuristics generally have greater exploration compared to single solution-based algorithms. Fourthly, multiple candidate solutions assist each other in GWO algorithm to avoid locally optimal solutions. 

The remainder of the work is organized as follows: in Section 2, models for extended target tracking are presented. Section 3 embeds the extended tracking problem into the GWO algorithm. Also, in this section, the detailed description and implementation of the GWO-TBD are presented. To evaluate the performance of the proposed method, four real scenarios and synthetic data are tested under various conditions in Section 4. Section 5 draws simple conclusions.

## 2. Preliminaries

### 2.1. Target Model

The extended target state ***ξ**_k_* at *k*-th scan is defined as the triplet ***ξ**_k_* = (*γ*, ***x**_k_*, ***X**_k_*) in [10]. Firstly, the random variable *γ* > 0 is the measurement rate that describes how many measurements the target, on average, generates per time scan. Secondly, ***x**_k_*′ = (***p**_k_*′, *v_k_*, *α_k_*)^T^∈ℝ^4^ is the kinematic state. ***p**_k_*′ describes the target’s position where ***p**_k_*′ = (*x_k_*′, *y_k_*′). *v_k_* denotes the velocity and *α_k_* represents the course of the targe. Finally, ***X**_k_* is the extension of the target and it describes the target’s size and shape. The target shape is assumed to be an ellipse because it is a good combination of an informative shape model and low computational complexity. The size of the target is denoted by the major axis *l*′ and minor axis *w*′ of the ellipse. The dynamic models and sensor measurement processes related to the state of target ***ξ**_k_* at *k*th scan are given by Equations (1) and (2), respectively:

(1) $$\xi_{k+1}=F(\xi_{k},\sigma )$$

(2) $${\left\{z\right\}}_{k}^{T}=H(\xi_{k},\omega )$$

where *F* (•) is the state propagation function and *H* (•) is the measurement function. Process noise *σ* and measurement noise *ω* are zero mean, white and uncorrelated Gaussian noise sequences. In (2), {***z***}*_k_*^T^ denotes the measurements of extended target at *k*th scan. |{***z***}*_k_*^T^| is the number of elements in {***z***}*_k_*^T^. Then, according to the Poisson distribution [21] it has:

(3) $$P(|{\left\{z\right\}}_{k}^{T}|=n|\gamma )=\frac{{\left(\gamma \right)}^{n}}{n!}\exp(\gamma )$$

The set of measurements generated by clutter is denoted by {***z***}*_k_*^C^. The set of measurements ***Z**_k_* obtained at time *k* is the collection of measurements generated by targets and clutters. Each measurement ***z**_k_* usually consists of a kinematic (position) measurement component (*x_k_^i^*, *y_k_^i^*) and a time stamp records the received time *t_k_^i^*:

(4) $$Z_{k}={\left\{z\right\}}_{k}^{T}\cup {\left\{z\right\}}_{k}^{C}=\{z_{k}^{i}|i=1,\ldots ,N_{k}\}=\{(x_{k}^{i},y_{k}^{i},t_{k}^{i})|i=1,\ldots ,N_{k}\}$$

The set of all the measurements in a time series is denoted by ***Z**^K^*, ***Z**^K^* = { ***Z**_k_* }*_k_*_= 1_*^K^* and the input of the GWO-TBD is just the points set ***Z**^k^*.

### 2.2. Problem Statement

The TBD algorithm is a method to improve the detection of weak targets by integrating their signal returns over multiple consecutive scans, i.e. estimating the state of targets at each scan ***ξ*** by measurement ***Z**^k^*. The optimal estimation which has a maximum likelihood is:

(5) $$\xi_{T}=\arg\max P(\xi |Z^{k});\xi =\{\xi_{k}{\}}_{k=1}^{K}$$

**Figure 1:**
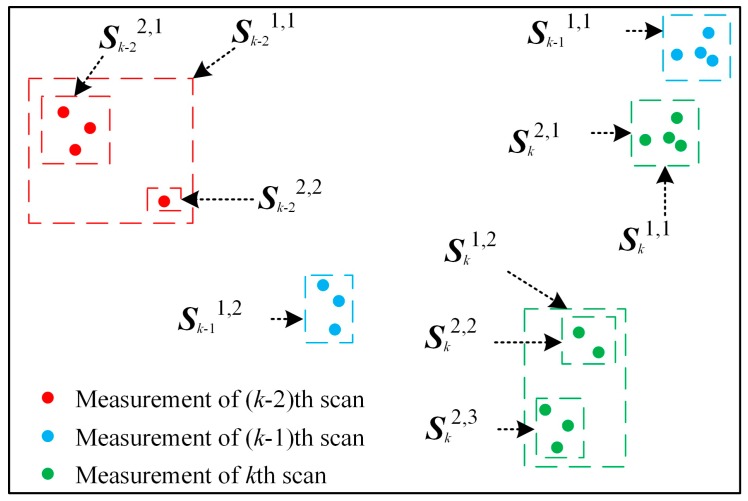
Diagram of alternative distance partitioning.

For the enormity of the solution space of ***ξ***, the estimation of the optimal solution ***ξ***_T_ is divided into two stages. Firstly, the measurements originated from the extended target are abstracted. According to the random matrix approach [22], the measurements of one scan should be clustered into sets. The measurements in a set are potentially generated by the extended target. Although two improved measurement partition methods are developed in [11,12], distance partitioning [1] is utilized here for its simplification and robustness. The sets of measurements partitioned by a single distance are represented by ***S**_k_*^1^, ***S**_k_*^1^, …, ***S**_k_^Mk^* where *M_k_* denotes the number of sets in *k*-th scan. One measurement must belong to only one set, i.e. (7):

(6) $$S_{k}^{i}=\{z_{k}^{i,j},j=1,\ldots ,\left|S_{k}^{i}\right|\}=\{(x_{k}^{i,j},y_{k}^{i,j},t_{k}^{i,j}),j=1,\ldots ,\left|S_{k}^{i}\right|\},$$

(7) $$S_{k}^{i}\cap S_{k}^{j}=\emptyset ,$$

where ***z**_k_^i,j^* means *j*-th point in *i*-th set at the *k*-th scan. Similarly, alternative distance partitioning can be obtained by multiple distances. The partition result of the *i*-th distance partitioning can be represented by ***S**_k_^i,^*^1^, ***S**_k_^i,^*^2^,…, $S_{k}^{i,M_{k}^{i}}$. The quantity of alternative partitions is represented by *M_k_*. Then, it has:

(8) $$Z_{k}=S_{k}^{i,1}\cup \;S_{k}^{i,2}\cup \ldots \cup S_{k}^{i,M_{k}^{i}};i=1,\ldots M_{k},$$

(9) $$S_{k}^{i.j}=\{z_{k}^{i,j,n},n=1,\ldots ,\left|S_{k}^{i,j}\right|\}=\{(x_{k}^{i,j,n},y_{k}^{i,j,n},t_{k}^{i,j,n}),n=1,\ldots ,\left|S_{k}^{i,j}\right|\}$$

where ***z**_k_^i,j,n^* means the *n*-th point in the *j*-th set under *i*-th partitioning distance in the *k*-th scan. *M_k_^i^* denotes the number of sets if the measurements are partitioned by the *i*-th distance in the *k*-th scan. Meanwhile, the quantity of partition in this scan is represented by *M_k_*, it also means the quantity of partition distance. The alternative distance partitioning can be illustrated by Figure 1. The measurements of *k*th scan (green points) can be clustered into three sets with a small distance and two sets with a larger distance. The diagram in Figure 1 also infers that the quantity of alternative partitions *M_k_* is alternative and determined by the spatial distribution of measurements.

Since, the measurements of an extended target can be described by a series of measurement sets, the association of some measurement sets can be regarded as a potential trajectory. In this context, the association equals a solution in SI algorithms and *s*th candidate solution can be represented by ***C**_s_*:

(10) $$C_{s}=\{S_{k}^{i,j},k=k_{s},\ldots ,k_{e}\}$$

**Figure 2:**
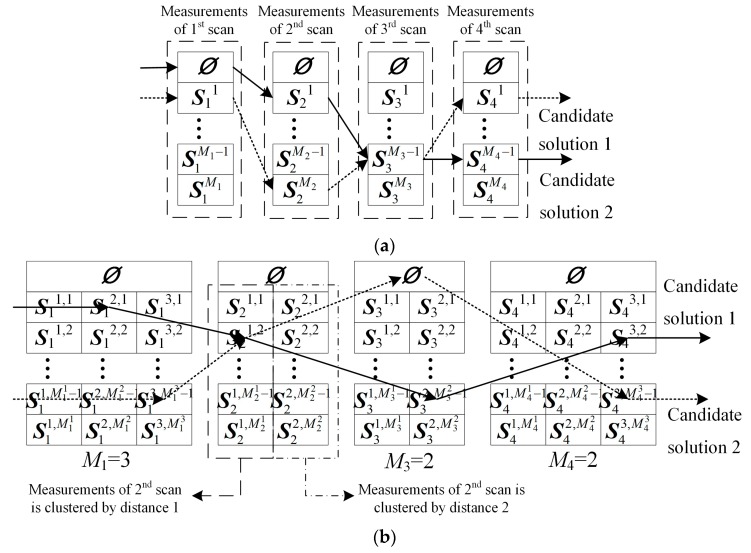
The schematic diagram of the candidate solution. (**a**) Candidate solution with a single distance threshold is applied to partition the measurements. (**b**) Candidate solution with alternative partitions are applied to partition the measurements.

Equation (10) assumes that the target is detected and lost at the *k*_s_-th and *k*_e_-th scan, respectively. Figure 2 is presented to further explain the structure of the candidate solution. Figure 2a,b showcase that a single distance and multiple distances are applied to partition the measurements. The measurement sets are associated by the black lines for forming the candidate solution 1. The dotted lines are corresponding to the association for the candidate solution 2. The dashed box in Figure 2a denotes the measurements of one scan. The dashed box in Figure 2b means a partition of the measurements in one scan. 

In Figure 2, *Ø* means an empty set. The probability of no measurements of the target is received by the sensor *P_m_* is then given as:

(11) $$P_{m}=1-(1-e^{-\gamma })\times P_{d}$$

This assumes that the target is detected with probability *P_d_* with a sensor. The weak target usually has a large *P_m_*. For example, *P_m_* is approximately equal to 0.374 when *γ* and *P_d_* equal 1 and 0.99 respectively. Naturally, empty set is reasonable to be selected as a measurement set in a solution. Then, the aim of this stage becomes finding the optimal solution ***C***_T_ by measurement set ***Z**^k^*:

(12) $$C_{T}=\arg\max P(C|Z^{k})$$

For instance, the optimal solution ***C***_T_ in Figure 1 can be the association of sets {***S**_k-_*_2_^2,1^, ***S**_k-_*_1_^1,2^, ***S**_k_*^2,3^}. 

Secondly, the optimal solution ***ξ***_T_ can be estimated by measurements of the extended target ***C***_T_ and a smoothing filter. In this work, an orthogonal least squares fit is applied for the ***ξ***_T_.

### 2.3. GWO Algorithm

The GWO algorithm is an adaptive metaheuristic search algorithm inspired by the hunting and searching behavior if wolves. In GWO, a complete wolf pack consists of alpha (*α*), beta (*β*), delta (*δ*), and omega (*ω*) wolves. The best wolves should be treated as *α*, *β*, and *δ* that assist other wolves (*ω*) in exploring more favorable regions of solution space. The alphas are the leaders of the pack, responsible for making decisions. The alphas’ decisions are dictated to the pack. The betas are subordinate wolves that help the alpha in decision making or other activities. The best candidates to be the alpha is mostly likely the betas. The omega wolves are the scapegoats of the pack, they have to submit to all the other dominant wolves. The deltas have to submit to alphas and betas, but they dominate the omegas [23]. The rank of the wolves equals the fitness of the solutions. According to the differences in the rank of the wolves, in order to have better knowledge about the potential location of prey, the alpha, beta and delta wolves are assumed to be the best, the second best and the third best candidate solution, respectively. 

In the conventional GWO, in order to mathematically model encircling behavior, the Equations (13)–(16) are used [19].

(13) $$\vec{D}=|\vec{B}\cdot C_{P}\left(t\right)-C|,$$

(14) $$C\left(t+1\right)=C_{P}\left(t\right)-\vec{A}\cdot \vec{D},$$

where *t* is iteration, $\vec{A}$ and $\vec{B}$ are random vectors, ***C*** indicates the vector of a grey wolf, and ***C**_P_* is location of the prey. The random $\vec{A}$ and $\vec{C}$ vectors are calculated as [19]:

(15) $$\vec{A}=2a\cdot r_{1}-\vec{a},$$

(16) $$\vec{B}=2{\vec{r}}_{2},$$

where the components of $\vec{a}$ are a temporal parameter and linearly decrease from 2 to 0 over the course of iterations, and *r*_1_, *r*_2_ are random vectors in [0, 1]. Grey wolves are capable of identifying the position of the prey and enclose it. Note that the fluctuation range of $\vec{A}$ is also decreased by $\vec{a}$. A smaller fluctuation range of $\vec{A}$ means a smaller step towards the optimal solution. More iteration is necessary but more likely to achieve the optimal solution. The first three best candidate solutions obtained can lead other hunters (including the omegas) to update their positions according to the position of the best search agents [24], so the states of the updated solutions of wolves are determined by Equation (17) [11]:

(17) $$C\left(t+1\right)=\frac{C_{1}+C_{2}+C_{3}}{3},$$

where *t* shows recent iteration and ***C**_1_*, ***C**_2_*, ***C***_3_ denote the final state of the updated solutions, they are defined as in Equations (18)–(20), respectively:

(18) $$C_{1}=C_{\alpha }-{\vec{A}}_{1}\cdot \left({\vec{D}}_{\alpha }\right),$$

(19) $$C_{2}=C_{\beta }-{\vec{A}}_{2}\cdot \left({\vec{D}}_{\beta }\right),$$

(20) $$C_{3}=C_{\delta }-{\vec{A}}_{3}\cdot \left({\vec{D}}_{\delta }\right),$$

where ***C**_α_*, ***C**_β_*, ***C**_γ_* denote the locations of alpha, beta, and delta wolves, respectively in the swarm at a given iteration *t*, ${\vec{A}}_{1}$, ${\vec{A}}_{2}$, ${\vec{A}}_{3}$ represent random vectors, and ${\vec{D}}_{\alpha }$, ${\vec{D}}_{\beta }$, ${\vec{D}}_{\delta }$ are defined using Equations (21)–(23), respectively:

(21) $${\vec{D}}_{\alpha }=\left|{\vec{B}}_{1}\cdot C_{\alpha }-C\right|,$$

(22) $${\vec{D}}_{\beta }=\left|{\vec{B}}_{2}\cdot C_{\beta }-C\right|,$$

(23) $${\vec{D}}_{\delta }=\left|{\vec{B}}_{3}\cdot C_{\delta }-C\right|,$$

where ${\vec{B}}_{1}$, ${\vec{B}}_{2}$, ${\vec{B}}_{3}$ are defined as representative random vectors. 

The updating of the parameter controls the tradeoff between exploration and exploitation in the grey wolf optimizer (GWO). The parameter a is linearly decreased in each iteration to range from 2 to 0 according to Equation (24):

(24) $$a=2\left(1-t\frac{1}{MaxIter}\right),$$

where *MaxIter* is the total number of iteration allowed for the optimization and *t* is the iteration number.

## 3. Methodology

### 3.1. Introduction of the GWO-TBD

In this section, the basic idea of the GWO-TBD method is presented. The input of the GWO-TBD are the three dimensional points during a period of time, such as the measurements of several successive frames. As presented in Equation (4), the three dimensional points include a two dimensional positional information and its measuring time. The output is a 3D-line that consists of 3D-points regarding the estimated location and time of the target. Two stages exist in the method, finding the measurements of the target and smoothing the trajectory. In stage one, a modified GWO is introduced to find the measurements and it is the most fundamental constituent of this GWO-TBD. The candidate solution here is an association which selects one measurement set in each scan. The formation of the candidate solution has been presented in Figure 2b. The pool of solutions is a *K* (number of scans) dimensional space. The quantity of candidate solution in theory equals:

(25) $$\prod_{k=1}^{K}(\sum_{i=1}^{M_{k}}M_{k}^{i}+1)$$

An exhaustive search for the optimal association of measurement set in so huge the space of solutions is unpractical. Therefore, a modified GWO is exploited in measurement selection for the optimal trajectory. The overview of the GWO-TBD is presented by following steps:

Step 1, measurements are clustered into sets, each set regards the measurements as potentially originated from the extended target, by the alternative partitioning approach in [1]. 

Step 2, the initial population (multiple solutions) is generated with the spatial relationship of measurements. 

Step 3, the fitness function of each candidate solution is calculated. The value of fitness function, by definition, is the probability that the candidate solution is the trajectory of an extended target. The global-best solution (*α* wolf), the second-best individual (*β* wolf) and the third-best individual (*γ* wolf) are estimated. 

Step 4, the position of each individual is updated by GWO. Some unappreciated solutions would be removed.

Step 5, tracklet fusion is conducted. Two tracklets may be combined to form a better trajectory. 

Step 6, the parameters in GWO-TBD is updated for better exploration and exploitation of candidate individuals.

Step 7, if stopping criterion is met, stop and output the best solution achieved so far, otherwise, go to Step 3. 

Step 8, an orthogonal least squares fit is applied on the best solution for a more smooth and accurate trajectory. This step is the stage two in the GWO-TBD. 

The individuals will evolve through the course of selection, fusion and updating iteratively. This is exactly what the GWO is introduced for. The accurate implementation of the GWO-TBD is presented in the following section. 

### 3.2. Implementation of the GWO-TBD

#### 3.2.1. Initial Population

It is desirable that the initial population be scattered uniformly over the feasible solution space, so that the algorithm can explore the whole solution space evenly. Meanwhile, some impossible solutions should be avoided. If two sets are generated by the same target, the spatial distance between the two sets should be smaller than the product of the time interval and the maximum velocity *V_max_*. It means that, two sets should not put in one candidate solution when Equation (26) holds:

(26) $$\sqrt{{\left({\bar{x}}_{k}^{a,b}-{\bar{x}}_{k+1}^{c,d}\right)}^{2}+{\left({\bar{y}}_{k}^{a,b}-{\bar{y}}_{k+1}^{c,d}\right)}^{2}}>({\bar{t}}_{k}^{a,b}-{\bar{t}}_{k+1}^{c,d})\times V_{\max}$$

(27) $${\bar{x}}_{k}^{a,b}=\frac{\sum_{n=1}^{\left|\;S_{k}^{a,b}\right|}x_{k}^{a,b.n}}{\left|S_{k}^{a,b}\right|};{\bar{y}}_{k}^{a,b}=\frac{\sum_{n=1}^{\left|S_{k}^{a,b}\right|}{\bar{y}}_{k}^{a,b.n}}{\left|S_{k}^{a,b}\right|};{\bar{t}}_{k}^{a,b}=\frac{\sum_{n=1}^{\left|\;S_{k}^{a,b}\right|}{\bar{t}}_{k}^{a,b.n}}{\left|S_{k}^{a,b}\right|}$$

Then the strategy to generate the initial population can be concluded as: associate the sets at different scans randomly and uniformly following the criterion in Equation (26). *N_p_* candidate solutions generated and the *i*-th candidate solution is denoted by parameter ***C**_i_* here. Selecting a larger *N_p_* means more calculation but taking the advantages of good stability and strong search ability:

(28) $$C_{i}=\{S_{k}^{i,j}|k=k_{s},\ldots ,k_{e}\}=\{\;C_{i}^{k_{s}},\ldots ,C_{i}^{k_{e}}\}$$

It is worth noting that the initial solution is merely a tracklet which starts at scan *k*_s_ and ends at *k*_e_. The current candidate solution equals a wolf in GWO. 

#### 3.2.2. The Fitness Function

The fitness function *F_C_*(***C**_a_*) is designed to estimate the probability that the candidate solution is the trajectory of a target. The definition of the *F_C_*(***C**_a_*) is:

(29) $$F_{C}(C_{a})=p_{s}\prod_{k=k_{s}}^{k_{e}}F_{S}(S_{k}^{i,j},C_{a})p_{e}$$

*P*_s_ and *P*_e_ denote the probability of a target is emerge and disappear, respectively. *F_S_*(***S**_k_^i,j^*, ***C**_a_*) means the fitness of a set ***S**_k_^i,j^*. It is designed inversely proportional to the difference between ***S**_k_^i,j^* and the estimated set of *k*th scan by the other sets in this candidate solution. Meanwhile, it has three parts corresponding to the triple state of an extended target (*γ*, ***x**_k_*, ***X**_k_*):

(30) $$F_{S}(S_{k}^{i,j},C_{a})=F_{\gamma }(S_{k}^{i,j},C_{a})F_{x}(S_{k}^{i,j},C_{a})F_{X}(S_{k}^{i,j},C_{a})$$

The three functions *F*_γ_(●), *F***_x_**(●) and *F***_X_**(●) represent the probability that the set ***S**_k_^i,j^* is generated by the target using the information of measurement rate, target position, target extension respectively. The measurement rate *γ* of extended target is assumed invariant. The estimated measurement rate $\hat{\gamma }$ equals the average number of the measurements in these sets:

(31) $$\hat{\gamma }=\frac{\sum_{t=k_{s},\ldots ,k_{e};t\neq k}\left|S_{t}^{i,j}\right|}{k_{e}-k_{s}}$$

Then the *F*_γ_(***C**_a_*, ***S**_k_^i,j^*) in Equation (30) can be estimated by:

(32) $$F_{\gamma }(S_{k}^{i,j},C_{a})=\frac{{\hat{\gamma }}^{\left|S_{k}^{i,j}\right|}}{\left|S_{k}^{i,j}\right|!}\exp(-\hat{\gamma })$$

An estimated location (${\hat{x}}_{t}^{i,j},{\hat{y}}_{t}^{i,j}$) can be obtained if the location of a target in other scans is given by smoothing filter in [25]:

(33) $$\left({\hat{x}}_{t}^{i,j},{\hat{y}}_{t}^{i,j})=F({\bar{x}}_{t}^{i,j},{\bar{y}}_{t}^{i,j},{\bar{t}}_{t}^{i,j}|t=k_{s},\ldots ,k_{e};t\neq k\right)$$

Then the *F***_x_**(***C**_a_*, ***S**_k_^i,j^*) in Equation (30) can be estimated by:

(34) $$F_{x}(S_{k}^{i,j},C_{a})=N(d|0,\sigma );d=\sqrt{{\left({\hat{x}}_{t}^{i,j}-{\bar{x}}_{t}^{i,j}\right)}^{2}+{\left({\hat{y}}_{t}^{i,j}-{\bar{y}}_{t}^{i,j}\right)}^{2}}$$

*N*(*d*;0, *σ*) denotes a Gaussian distribution defined over the variable *d* with mean 0 and covariance *σ*. As to the target extension *F***_X_**(***C**_a_*, ***S**_k_^i,j^*), extension state ***X**_k_* is described an inverse Wishart probability distribution function (PDF). ***IW**_d_*(***X***; *v*, *V*) in Equation (35) denotes an inverse Wishart pdf defined over the matrix ***X*** with degrees of freedom *v* and parameter matrix *V*:

(35) $$F_{X}(S_{k}^{i,j},C_{a})=IW_{d}(X_{k}^{i,j};v_{k},V_{k})=\frac{2^{-\frac{v_{k}-d_{X}-1}{2}}|V_{k}{|}^{-\frac{v_{k}-d_{X}-1}{2}}}{\Gamma_{d_{X}}(\frac{v_{k}-d_{X}-1}{2})|X_{k}{|}^{\frac{v_{k}}{2}}}\exp(\mathrm{Tr}(-\frac{1}{2}{\left(X_{k}\right)}^{-1})V_{k})$$

where Γ*_d_*(●) is the multivariate gamma function, *d***_X_** means the dimension of the matrix ***X*** and Tr(•) denotes the trace of a matrix. Degrees of freedom *v* and parameter matrix *V* can be estimated by the measurement sets $\left\{S_{t}^{i,j}|t=k_{s},\ldots ,k_{e};t\neq k\right\}$ by [26]. The lower and upper limit of *F_S_*(***S**_k_^i,j^*, ***C**_a_*), the fitness of set ***S**_k_^i,j^*, are 0 and 1 respectively. Then, the fitness of all *N_p_* candidate solutions is calculated by Equation (29). The *α*, *β*, *δ* solutions based on fitness ***C**α*, ***C**_β_*, ***C**_δ_* can be found.

#### 3.2.3. Selection, Abruption, and Fusion

Selection operates in a way such that each and every member of the population has a chance to be selected, but the better the fitness value of a candidate solution *F_C_*(***C**_i_*) the more chance of being selected it will have. Some unappreciated solutions can be removed. 

Then abruption and fusion are performed to find the updated grey wolf positions. Tracklet abruption and fusion mimic the reproduction in the nature. Given parent wolves, selected from the survival, child wolves which inherit the merits of parents are generated. Here, some better solutions maybe generated by existing candidate solutions. In abruption, unappreciated association of measurement sets should be broken up. The fitness function of sets *F_S_*(***S**_k_^i,j^*, ***C**_a_*) of each candidate solution has been calculated in the last step. A candidate solution can be broken up at the measurement set which has the lowest fitness function if Equation (36) holds:

(36) $$p_{s}\prod_{k=k_{s}}^{k_{e}}F_{S}(S_{k}^{i,j},C_{a})p_{e}<p_{s}\prod_{k=k_{s}}^{k_{b}}F_{S}(S_{k}^{i,j},C_{a})p_{e}\times p_{s}\prod_{k=k_{b}+1}^{k_{e}}F_{S}(S_{k}^{i,j},C_{a})p_{e}$$

Equation (36) assumes that the measurement set $S_{k_{b}}^{i,j}$ has the lowest fitness function and the $S_{k_{b}}^{i,j}$ divides the candidate solution ***C**_a_* into two shorter tracklets ***C**_a_*_1_ and ***C**_a_*_2_:

(37) $$\underset{C_{a}}{\underset{︸}{\left\{S_{k}^{i,j},k=k_{s},\ldots ,k_{e}\right\}}}\underset{\mathrm{Abruption}}{\Rightarrow }\underset{C_{a1}}{\underset{︸}{\left\{S_{k}^{i,j},k=k_{s},\ldots ,k_{b}\right\}}}\&\underset{C_{a2}}{\underset{︸}{\left\{S_{k}^{i,j},k=k_{b+1},\ldots ,k_{e}\right\}}}$$

Then, solution fusion is performed. Two shorter tracklets ***C**_a_*_1_ and ***C**_a_*_2_ can be combined together to form a longer one if Equation (38) holds:

(38) $$\underset{C_{a}}{\underset{︸}{p_{s}\prod_{k=k_{s}}^{k_{e}}F_{S}(S_{k}^{i,j},C_{a})p_{e}}}>\underset{C_{a1}}{\underset{︸}{p_{s}\prod_{k=k_{s}}^{k_{b}}F_{S}(S_{k}^{i,j},C_{a})p_{e}}}\times \underset{C_{a2}}{\underset{︸}{p_{s}\prod_{k=k_{b}+1}^{k_{e}}F_{S}(S_{k}^{i,j},C_{a})p_{e}}}$$

**Figure 3:**
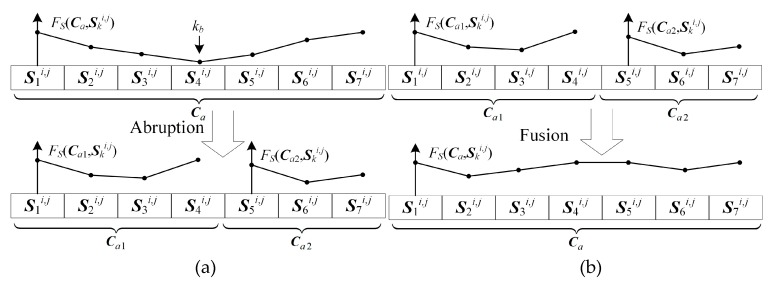
(**a**) An example on the tracklet abruption. (**b**) An example on the tracklet fusion.

The fusion can be accelerated and the abruption can be restrained by setting a larger value of *p_e_* and *p_s_*. Two examples on abruption and fusion are given in Figure 3. 

The *α*, *β*, *δ* solutions are free from the selection, abruption, and fusion.

#### 3.2.4. Exploration

In this step, the position of the omega wolves is updated towards the alpha, beta and delta wolves. The omega solutions would be further optimized towards the best three solutions. Different with the updating equation of continuous GWO in Equation (17), the main updating equation here can be formulated in Equation (39):

(39) $$C\left(t+1\right)=Crossover(C_{1},C_{2},C_{3},C_{b})$$

where *Crossover*(***C***_1_, ***C***_2_, ***C***_3_, ***C**_b_*) is a suitable crossover between solutions ***C***_1_, ***C***_2_, ***C***_3_ and ***C***_1_, ***C***_2_, ***C***_3_ are discrete vectors representing the effect of wolf move towards the alpha, beta and delta grey wolves in order. ***C***_b_ is the candidate solution which is updating its selection of measurement sets. ***C***_1_, ***C***_2_, ***C***_3_ are calculated using Equations (40), (42), and (43), respectively:

(40) $$C_{1}^{d}=\{\begin{array}{ll} C_{\alpha }^{d} & \mathrm{if}\;(F_{S}(S_{d}^{i,j},C_{\alpha })+cstep_{\alpha }^{d}>1)\\ C_{b}^{d} & \mathrm{otherwise} \end{array},$$

where ***C**_α_^d^* is the selection of the alpha wolf in the *d*-th scan. Parameter *cstep_α_^d^* is the continuous valued step size for *d*-th scan and can be calculated using sigmoidal function as in Equation (41):

(41) $$cstep_{\alpha }^{d}=\frac{1}{1+e^{-10(A_{1}^{d}D_{\alpha }^{d}-0.5)}},$$

where *A_α_^d^*, *D_α_^d^* are calculated using Equations (15) and (21) in original GWO algorithm. Similarly, it has:

(42) $$C_{2}^{d}=\{\begin{array}{ll} C_{\beta }^{d} & \mathrm{if}\;(F_{S}(S_{d}^{i,j},C_{\beta })+cstep_{\beta }^{d}>1)\\ C_{b}^{d} & \mathrm{otherwise} \end{array}$$

(43) $$C_{3}^{d}=\{\begin{array}{ll} C_{\delta }^{d} & \mathrm{if}\;(F_{S}(S_{d}^{i,j},C_{\delta })+cstep_{\delta }^{d}>1)\\ C_{b}^{d} & \mathrm{otherwise} \end{array}$$

**Figure 4:**
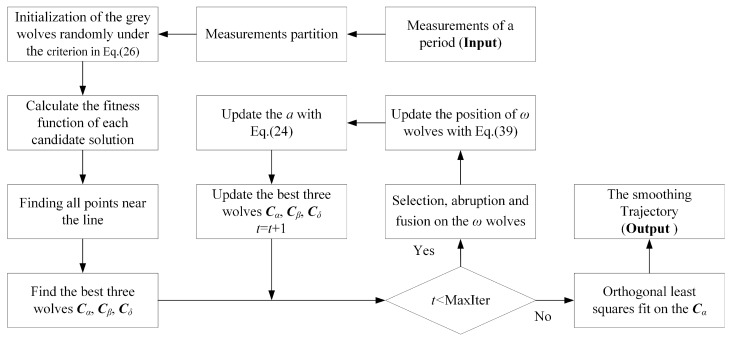
The roadmap of the GWO-TBD method.

A simple stochastic crossover strategy is applied each scan to crossover ***C***_1_, ***C***_2_, ***C***_3_ solutions as shown in Equation (44):

(44) $$C_{b}^{d}(t+1)=\{\begin{array}{ll} C_{1}^{d} & \mathrm{if}\;(rand<\frac{1}{3})\\ C_{2}^{d} & \mathrm{if}\;(\frac{1}{3}\leq rand<\frac{2}{3})\\ C_{3}^{d} & \mathrm{otherwise} \end{array}$$

where *rand* is a random number drawn from uniform distribution in the range [0, 1]. The roadmap of the GWO-TBD is presented in Figure 4 for better description.

**Figure 5:**
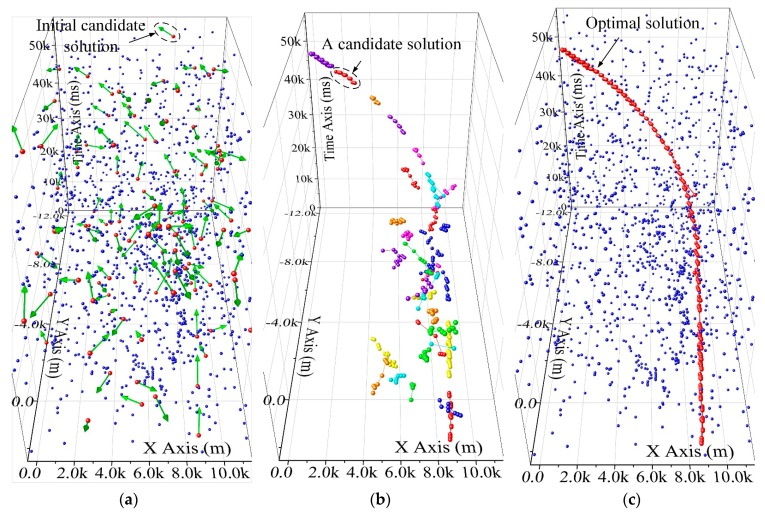
The utilization of the GWO-TBD in a real scenario. (**a**) The initial candidate solutions. (**b**) The candidate solutions in the iteration. (**c**) The optimal solution.

Meanwhile, the diagram of utilizing the GWO-TBD in a real scenario is presented in Figure 5. In Figure 5a,c, the measurements are represented by the blue points. The blue points are showcased in a 3-dimensional Cartesian coordinate system. Values of points on thhe x-y axes represent the location of the target, while the value of the third axis denotes the measuring time. In Figure 5a, the initial candidate solutions are represented by the green arrows, each of which is associating two measurement sets in different scans. The red point on the tail of the green arrows denotes the centroid of the measurement set which is used to form the initial tracklet.

In Figure 5b the candidate solutions are represented by the tracklet in different colours. The optimal solution in Figure 5c showcases that the points of a maneuvering extended target can be extracted well after several generations. 

## 4. Experimental

### 4.1. Synthetic Result

**Figure 6:**
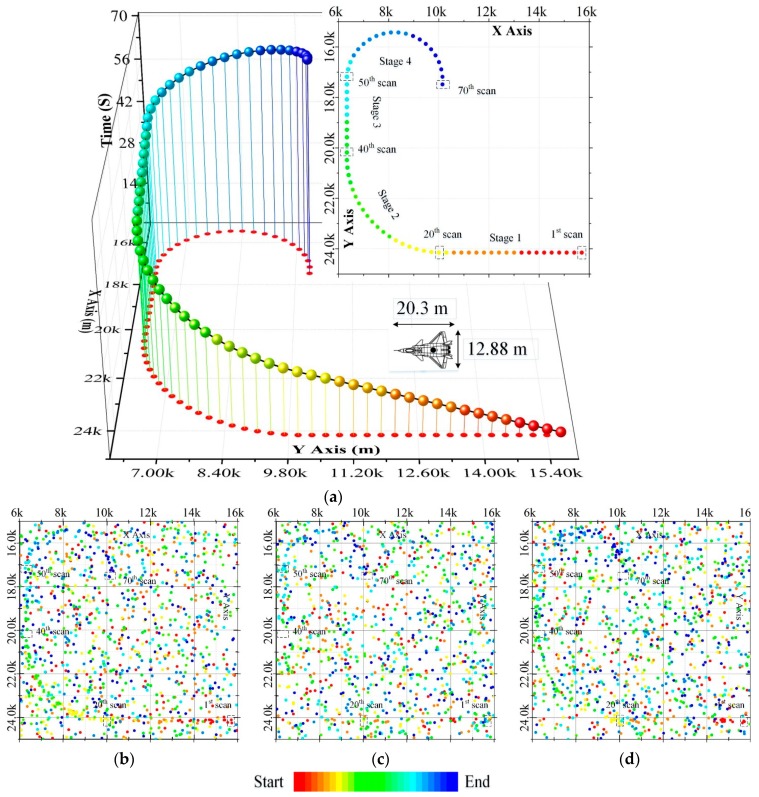
The synthetic data in this work. (**a**) The trajectory of the simulated target. (**b**) The synthetic measurements of scenario 2. (**c**) The synthetic measurements of scenario 3. (**d**) The synthetic measurements of scenario 4.

In order to evaluate the performance of the GWO-TBD algorithm, 200 Monte Carlo numerical simulations are performed on an Intel Core I7-4790 3.6 GHz CPU, equipped with 4 GB RAM in the MatLab R2016a environment. The specific trajectory of an airplane travelling at a constant speed is presented in Figure 6a. The airplane flies on a straight line at the beginning and maneuvers during the 21-st scan to the 40-th scan and during the 51-st scan to the 70-th scan. The whole trajectory can be divided into four parts, stage 1 (straight line, 1-st–20-th scans), stage 2 (maneuvering, 21-st–40-th scans), stage 3 (straight line, 31-st50-th scans) and stage 4 (highly maneuvering, 51-st–70-th scans). It is worth noting that the proposed approach is designed for detecting 2-dimensional trajectories of the target, the third axis in Figure 6a is the measuring time, not the altitude of the target. 

In this work, six scenarios are considered to validate both the accuracy and robustness of the algorithms, the measurement rate of targets, the measurement noise and the false alarm rate are varied in each scenario. The detailed parameters of the scenarios are presented in Table 1. It is worth noting that the probability of no measurements generated by a target in a scan equals 36.78%, 13.53% and 1.83% when the measurement rate equals 1, 2 and 4, respectively. It is hard to detect or track an extended target if no measurements are generated by it. Meanwhile, a larger measurement noise means a larger localisation error. Larger measurement noise and dense clutter would significantly deteriorate the tracking performance. The parameters of the radar in this work is patched in Table A1.

Figure 6b–d show the synthetic data in scenario 2, scenario 3 and scenario 4. It is difficult to detect the targets in these scenarios with the naked eyes. The parameters of the radar in this work is presented in Table A1 of the Appendix A.

In this work, both the ET-PHD filter-based approaches and track-before-detect methods are compared with the GWO-TBD. In the category of PHD approaches, the distance partition method [1], the ART partition method [11] and the AP partition method [12] are combined with the ET-PHD [1] respectively. In the category of TBD approaches, the 3DHT-TBD [10] and the 4DHT-TBD [9] are used. Parameters, such as the false alarm rate, measurement rate, initial state of the extended target, are fed to the PHD filter before its iteration. The optimal sub-pattern assignment (OSPA) distance [27] is used for evaluating the performance of the algorithms. The OSPA distance between the positions of *n* targets ***T*** *=* { ***T***_1_, ***T***_2_,…, ***T**_n_*} and the estimated positions ***p*** = { ***p***_1_, ***p***_2_,…, ***p**_n_*} in each scan can be calculated by:

(45) $$\mathrm{OSPA}(T,p)=\{\begin{array}{l} D_{p,c}(T,p),m>n\\ D_{p,c}(p,T),m\leq n \end{array}$$

(46) Dp,c(T,p)=(1n(minκ∈Ω∑i=1m(dc(Ti,pκ(i)))p+(n−m)cp))1p,m≤n

Ω represents the set of permutations of length *m* with elements taken from ***T***. The cut-off value *c* and the distance order *p* of OSPA distance are set as *c* = 150 and *p* = 1 in this work. Note that the cut-off parameter *c* determines the relative weighting given to the cardinality error component against the localisation error component. Smaller values of *c* tend to emphasize localisation errors and vice versa. 

**Figure 7:**
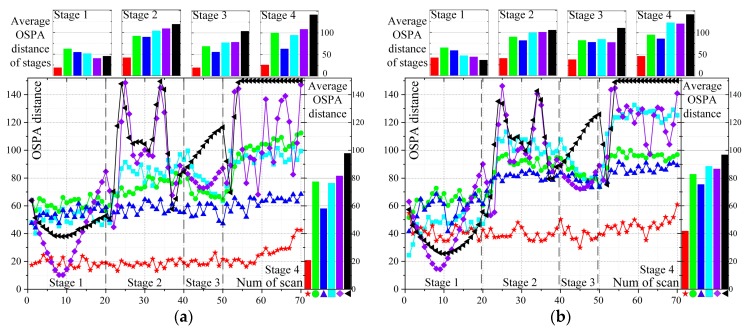

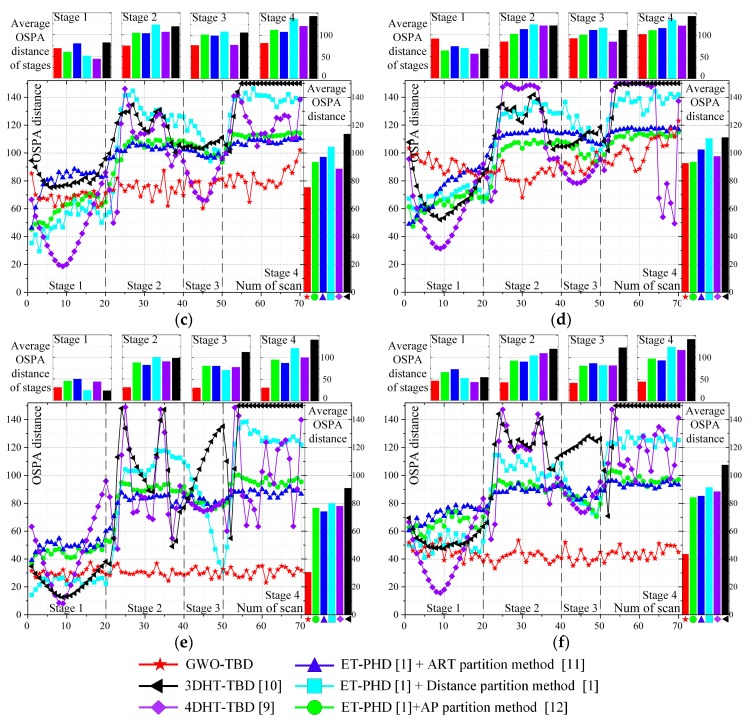
The OSPA distance of six scenarios at each scan. (**a**–**f**) correspond to scenario 1–6.

The results of the six algorithms at each scan are presented in Figure 7. Figure 7a corresponds to scenario 1, and so on. A smaller OSPA distance means a better tracking performance. In stage 1, the performance of the GWO-TBD is similar to the others. In stage 1 of scenario 4, the GWO-TBD is worse than the others. This is mainly because the initial state of the extended target is given in the ET-PHD filters. The Hough Transformation (HT)-based methods are intentionally designed for straight line detection. Meanwhile, the measurement rate of scenario 4 equals 1. With GWO-TBD it is hard to find the optimal trajectory because merely a few points are generated by the target. In the maneuvering stages, (stage 2 and stage 4), the performance of the ET-PHD filters and HT-based methods is greatly deteriorated. Especially in the stage 4, the HT-based methods could barely detect the target. However, there was almost no effect of maneuvering on the tracking of GWO-TBD. In stages 2 and 4 of all scenarios, the GWO-TBD performance is superior to the others. In stage 3, the ET-PHD filters are inferior to the GWO-TBD because the target is lost in stage 2 and detecting the trajectory in such scenarios is difficult.

The HT-based methods can obtain a better performance only in scenario 4 because of the deterioration caused by the low measurement rate in the GWO-TBD. In general, the average OSPA distance of the GWO-TBD is less than those of the others in all scenarios. The detailed values of the OSPA distance are listed in Table A2. The lowest OSPA distance in each scenario is emphasized in boldface. The measurement noise of scenario 2 is smaller than that of scenario 3. The comparison between the two scenarios infers that a lower OSPA distance can be achieved under a low measurement noise because the points are more centralized. Similarly, a comparison between scenario 2 and scenario 6 showcases that the higher the false alarm rate, the lower the tracking performance.

The performance of ET-PHD filters is related to the parameters of the scenario and movement of targets. With ET-PHD filters it is hard to achieve a satisfying result when the measurement noise or the false alarm rate is high, and when the target is weak or maneuvering. The measurement noise and the false alarm rate have little influence on the two HT-based methods and the performance would be greatly deteriorated when the target is maneuvering. The GWO-TBD can cope with the difficulties and is superior to the others in almost all the stages and scenarios (except stage 1 of scenario 4). 

Meanwhile, the parameters in the ET-PHD filters, such as the measurement rate and false alarm rate, have been set to fit the simulated data of each scenario. Table A3 in the Appendix A showcases the parameter values in several scenarios. It infers that the values of parameters in the ET-PHD are various in different scenarios. However, in the GWO-TBD, similar to the 3DHT-TBD, much fewer parameters are necessary to be given an appropriate value before the iteration. Fewer parameters allow the GWO-TBD more flexibility in use. Parameter values of the 3DHT-ET-TBD and the 4DHT are also presented in Table A4 of the Appendix A. The result infers that the GWO-TBD outperforms the others, especially when the target is maneuvering and little prior information is necessary.

### 4.2. Results with Real Data

**Figure 8:**
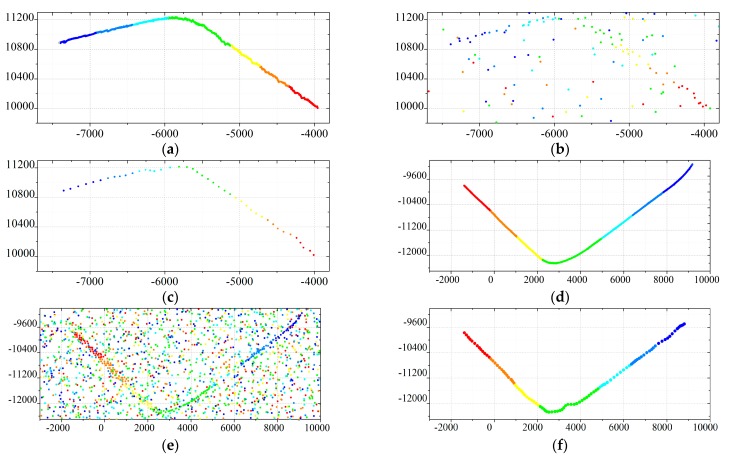

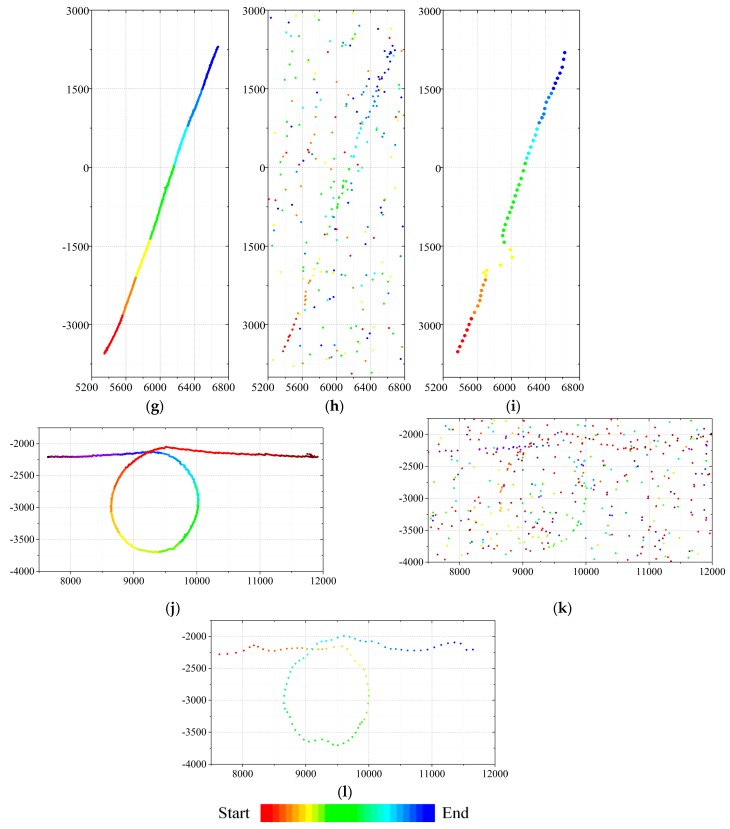
(**a**) Trajectory obtained by GPS in the scenario 1. (**b**) Measurements of the scenario 1. (**c**) Trajectory obtained by the GWO-TBD algorithm in the scenario 1. (**d**) Trajectory obtained by GPS in the scenario 2. (**e**) Measurements of the scenario 2. (**f**) Trajectory obtained by the GWO-TBD algorithm in the scenario 2. (**g**) Trajectory obtained by GPS in the scenario 3. (**h**) Measurements of the scenario 3. (**i**) Trajectory obtained by the GWO-TBD algorithm in the scenario 3. (**j**) Trajectory obtained by GPS in the scenario 4. (**k**) Measurements of the scenario 4. (**l**) Trajectory obtained by the GWO-TBD algorithm in the scenario 4.

To evaluate the performance of the proposed algorithm further, we conduct an experiment using an air surveillance radar located in a general airport of Pucheng City, ShannXi Provience, China. Acquisition of the radar data was performed in January, 2016. The real tracks of the targets are obtained by the Global Positioning System (GPS) in the airplane. The four real trajectories obtained by GPS are presented in Figure 8a,d,g,j. 

**Figure 9:**
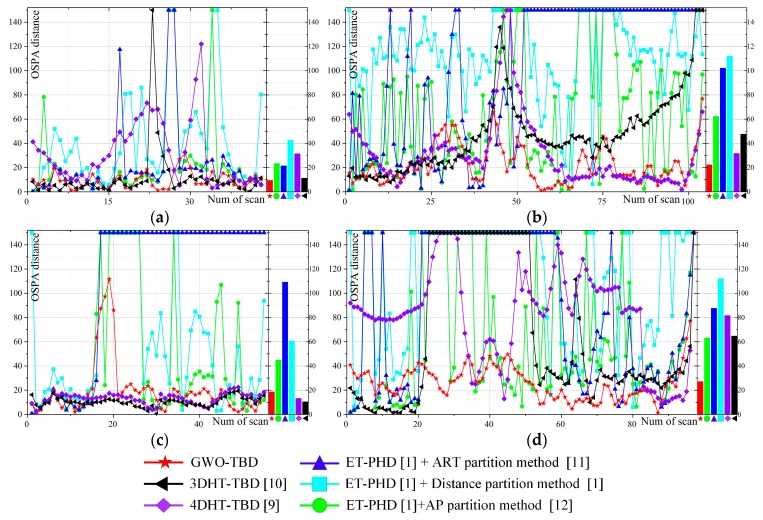
The OSPA distance of the four real scenarios. (**a**–**d**) correspond to real scenarios 1–4.

The colored curves represent the movement of the target in a Cartesian coordinate system. The measuring time of the target is represented by color from red to blue. Red and blue denote the starts and ends of the trajectories respectively. The measurements of the four scenarios are presented in Figure 8b,e,h,k. The measurement rate of the airplane is time varying and no measurements are generated by the airplane in some scans. Some clutter arise randomly in the surveillance area. Then, the results of the GWO-TBD are also shown in Figure 8c,f,i,l. The other methods are also applied using the real data. The initial state of the target in the ET-PHD filters is set to the correct values obtained by GPS. Actually, accurate values of the measurement rate and clutter rate are unknown. To achieve a better performance, the parameters of the ET-TBD method are different in different real scenarios. The specific values of the parameters can be found in Table A3. The OSPA distance of the four real scenarios is presented in Figure 9. Figure 9 infers that the OSPA distance of the GWO-TBD is much lower than the others, especially when the target is maneuvering. The ET-PHD filters substantially deteriorated when no points are generated by the target or the target is maneuvering. It is worth noting that the two HT-based methods are superior to the others in scenario 3, mainly due to the fact that the target is moving in a straight line and no points are generated by the target in eight successive scans (14-th–21-st scan). In the other three real scenarios where the target is maneuvering in some scans, the GWO-TBD are significantly outperformed the other methods. Especially in the 20-th–60-th scans of scenario 4, almost only the GWO-TBD works well in tracking such a weak maneuvering extended target. Comparison between scenario 4 and scenario 1 showcases that a lower measurement rate deteriorates the performance sharply because the target is hard to be detected when few measurements is originated by it. The OSPA distance of the four scenarios is also patched in Table A5 of the Appendix A.

Based on the experiment and analysis above, we can safely say that the GWO-TBD is more engineering friendly and better in detection and tracking performance.

## 5. Conclusions

In this article, the GWO was implemented to track and detect an extended target in a radar system. The algorithm was able to find the optimal association of measurement sets among the multiple scans. Targets can be well detected and tracked with the GWO-TBD. It is superior to the existing methods, especially when the extended target signal is weak or the target is maneuvering. Meanwhile, far less prior information is necessary before the iteration of the GWO-TBD, such as clutter rate of the surveillance area, extension and initial position of targets. Experiment infers that the GWO-TBD is better in performance and more practical in the real world. However, some limitations still exist. The GWO-TBD only copes with one target at a time. In multiple target tracking scenarios, the targets can be well detected one by one when the targets are far away from each other. The performance would be deteriorated if several maneuvering extended targets are closely distributed because several optimal solutions will exist in this scenario simultaneously. We would like to develop more approaches which can be used to detect multiple closely maneuvering extended targets from strong clutter in our later work.

## Figures and Tables

**Table 1:** The parameters of the scenarios.

Scenario	Measurement Rate γ	Measurement Noise (m)	Number of Clutter Per Square (1/m^2^)
Scenario 1	2	10	2 × 10^−7^
Scenario 2	2	50	2 × 10^−7^
Scenario 3	2	100	2 × 10^−7^
Scenario 4	1	50	2 × 10^−7^
Scenario 5	4	50	2 × 10^−7^
Scenario 6	2	50	4 × 10^−7^

## Appendix A

**Table A1:** Parameters of the radar.

Parameter	Value
3 dB azimuth beam width	1.17°
Angular Precision	0.0439°
Range Resolution	24(m)
Number of range bin	8192
Scanning cycle of radar	2(s)
Rotating speed of antenna	180(°/s)

**Table A2:** OSPA distance of synthetic data.

		S1	S2	S3	S4	Ave		S1	S2	S3	S4	Ave
GWO-TBD	Scenario 1	**18.7**	**18.2**	**19.8**	**26.2**	**20.8**	Scenario 4	91.2	**84.3**	91.8	**102.5**	**92.5**
ET-PHD [1] + AP [12]		62	74.3	68.8	99.8	77.3		63.8	101.7	100	111.5	93.4
ET-PHD [1] + ART [11]		54.4	57.6	55.8	63.1	58		73.6	112.9	110.9	116.4	102.4
ET-PHD [1] + Distance [1]		51.2	82.6	77.4	94.5	76.3		69.1	123.6	115.8	135.6	110.3
4DHT-TBD [9]		40.1	97.5	78.6	108.3	81.5		**56.2**	121.2	**83.9**	121.9	97.5
3DHT-TBD [10]		45.2	103.4	103.7	141.7	97.8		68	121.3	110.9	144.1	111.1
GWO-TBD	Scenario 2	41.3	**39.9**	**38.1**	**46**	**41.8**	Scenario 5	30.2	**30.4**	**30.5**	**30.7**	**30.5**
ET-PHD [1] + AP [12]		64.2	89.2	82.3	95.1	82.8		44.9	87.5	80.7	95.1	76.5
ET-PHD [1] + ART [11]		57.9	81	78.2	86.2	75.5		49.7	81.7	80.8	87.4	74.1
ET-PHD [1] + Distance [1]		45.8	98.7	84.7	123	88.5		23.4	99.5	70.9	121.4	79.9
4DHT-TBD [9]		43.3	100.3	77.8	120.7	86.6		43.5	90	78	100.2	77.9
3DHT-TBD [10]		**35.8**	104.9	110.9	142.2	96.7		**22.8**	97.8	112.7	141	90.9
GWO-TBD	Scenario 3	68.7	**74.6**	**76.8**	**81.9**	**75.3**	Scenario 6	45.3	**41.7**	**41.4**	**44.5**	**43.5**
ET-PHD [1] + AP [12]		60.2	104	101.4	112.2	93.4		65.2	91.6	80.9	97.3	84.2
ET-PHD [1] + ART [11]		79.8	103.1	98.8	107.5	97.1		71.9	89.3	86.8	93.4	85.2
ET-PHD [1] + Distance [1]		51.3	122.7	107.9	137.3	104.4		51.2	103.5	81.9	124.1	91.4
4DHT-TBD [9]		**44.3**	106.3	77.5	120.9	88.6		**42.4**	108.7	81.8	117.3	88.4
3DHT-TBD [10]		81.9	119	105.9	143.9	113.6		53.6	118.9	122.9	142.2	107.5

**Table A3:** Parameter values used in ET-PHD filter.

	Measurement Rate *γ*	Probability of Detection and Survival	Covariance of Systematic Error	Covariance of Measuring Error (*m*,°)	Number of Clutter Per Square (1/*m*^2^)
Scenario 1	2	[0.99, 0.99]	10	[20, 1.17]	2 × 10^−7^
Scenario 4	1	[0.99, 0.99]	50	[20, 1.17]	2 × 10^−7^
Scenario 6	2	[0.99, 0.99]	50	[20, 1.17]	4 × 10^−7^
Real scenarios 1, 3	2	[0.99, 0.99]	50	[20, 1.17]	2 × 10^−7^
Real scenario 2	2	[0.99, 0.99]	50	[20, 1.17]	3 × 10^−7^
Real scenario 4	1	[0.99, 0.99]	50	[20, 1.17]	2 × 10^−7^

**Table A4:** Parameter values used for simulations and real data.

Parameters in the 3DHT-ET-TBD		Parameter values used in the 4DHT	
Number of bins in X axis	100	Number of bins in X axis	100
Width of bins in X axis (m)	160	Width of bins in X axis (m)	160
Number of bins in Y axis	100	Number of bins in Y axis	100
Width of bins in Y axis (m)	160	Width of bins in Y axis (m)	160
Minimum vote count	15	Minimum vote count	15
Length of sliding window	7	Length of sliding window	7
Number of bins in 3D direction	541	Number of bins in velocity	60
		Width of bins in velocity (m/s)	15
		Number of bins in course	90
		Width of bins in course (°)	4

**Table A5:** OSPA distance of real data.

	Scenario 1	Scenario 2	Scenario 3	Scenario 4
GWO-TBD	**9.41**	**22.31**	18.73	**27.14**
ET-PHD [1] + AP [12]	23.33	62.27	44.85	63.12
ET-PHD [1] + ART [11]	21.49	102.1	109.25	87.62
ET-PHD [1] + Distance [1]	42.59	112.08	60.69	112.16
4DHT-TBD [9]	31.49	31.68	13.3	81.7
3DHT-TBD [10]	11.28	47.62	**10.45**	64.72
